# Supplementary material for: Untargeted and Targeted LC-MS/MS Based Metabolomics Study on In Vitro Culture of Phaeoacremonium Species
Source: J Fungi (Basel). 2022 Jan 6;8(1):55. doi: 10.3390/jof8010055 (PMC8780456; doi:10.3390/jof8010055)
Supplement: Supplementary file 1 [file jof-08-00055-s001.zip › jof-1501452-supplementary.pdf]

Untargeted and targeted LC-MS/MS based metabolomics study on *in vitro* culture of *Phaeoacremonium* species

Pierluigi Reveglia[1], Maria Luisa Raimondo[2], Marco Masi[3], Alessio Cimmino[3], Genoveffa Nuzzo[4],Gaetano Corso[1], Antonia Carlucci[2*], Angelo Fontana[4,5], Antonio Evidente[3*]

[1]Department of Clinical and Experimental Medicine, University of Foggia, Viale Pinto 1, 71121 Foggia, Italy

[2]Department of Agricultural Sciences, Food, Natural Resources and Engineering, Via Napoli 25 - 71122 Foggia, Italy

[3]Department of Chemical Sciences, University of Napoli Federico II, Complesso Universitario Monte Sant'Angelo, Via Cintia 4, 80126 Napoli, Italy.

[4] Institute of Bio-Molecular Chemistry, Consiglio Nazionale delle Ricerche (ICB-CNR), Via Campi Flegrei 34, 80078 Pozzuoli, Italy;

[5] Laboratory of Bio-Organic Chemistry and Chemical Biology, Department of Biology, University of Napoli "Federico II", Via Cupa Nuova Cinthia 21, 80126 Napoli, Italy

* Corresponding authors

Supplementary material

Figure S1. (**a**) XIC of MRM transitions of scytalone (**1**); (**b**) XIC of MRM transitions of isosclerone (**2**); (**c**) selected MRM transitions for scytalone (**1**); (**d**) selected MRM transitions for isosclerone (**2**).

Figure S2. Hierarchical Clustering result shown as dendrogram. In red the extracts at unmodified pH (UpH), in green the extracts after acidicifcation (pH 2) with formic acid.

Table S1. Calibration curves, correlation coefficient (r), imprecision, inaccuracy, LOD and LOQ value for scytalone (**1**) and isosclerone (**2**) quantification.

Table S2. Within-day imprecision (CV%) and inaccuracy (%) of results obtained by measuring two QC levels 6 times within one day (n=6).

Table S3. Between-day imprecision (CV%) and inaccuracy (%) of results obtained by measuring two QC levels once a day for five days (n=5).

Table S4. Workflow parameters Mzmine 2.

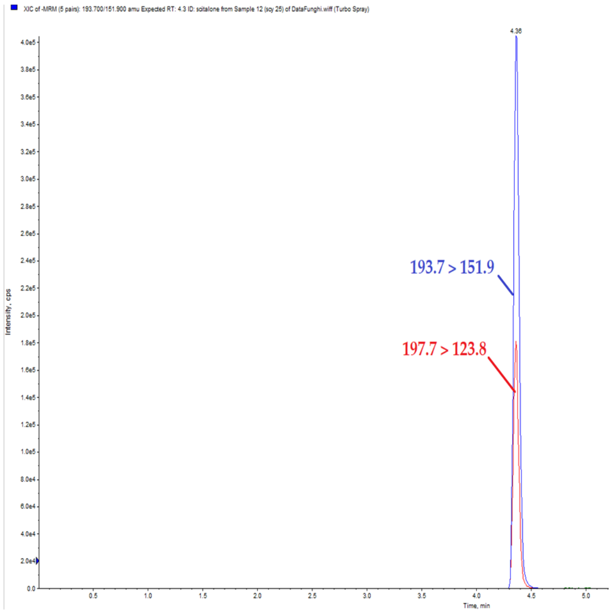

(**a**)

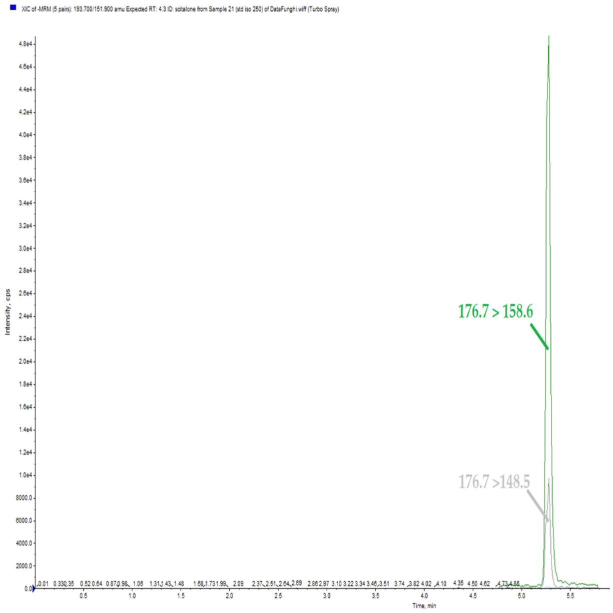

(**b**)

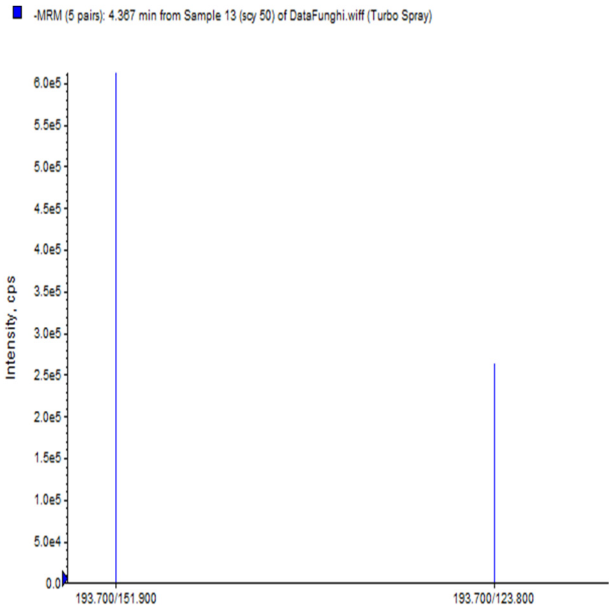

(**a**)

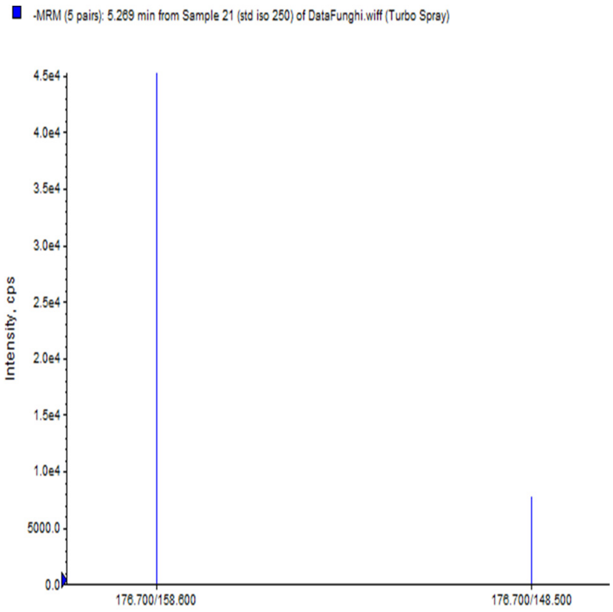

(**b**)

Figure S1. (**a**) XIC of MRM transitions of scytalone (**1**); (**b**) XIC of MRM transitions of isosclerone (**2**); (**c**) selected MRM transitions for scytalone (**1**); (**d**) selected MRM transitions for isosclerone (**2**).

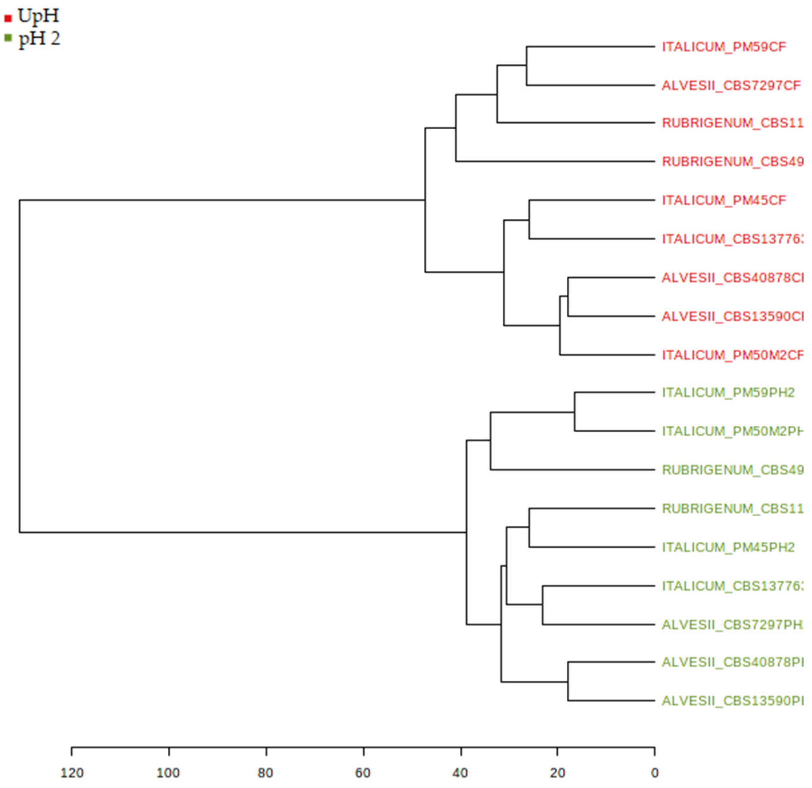

Figure S2. Hierarchical Clustering result shown as dendrogram. In red the extracts at unmodified pH (UpH), in green the extracts after acidicifcation (pH 2) with formic acid

Table S1. Calibration curves, correlation coefficient (r), imprecision, inaccuracy, LOD and LOQ value for scytalone (**1**) and isosclerone (**2**) quantification.

P	LOD	LOQ	Standard concentrations (µg/mL)	1	5	10	15	25
scytalone (**1**)	0.01	0.52	Mean	0.97	5.16	11.10	15.49	24.87
y = 0.23380 x + 0.02331			Imprecision[a]	0.9	9.5	3.0	1.5	5.7
r = 0.99904			Inaccuracy[b]	-2.6	3.2	11.0	3.3	-0.5
			**Standard concentrations (µg/mL)**	**15**	**30**	**60**	**150**	**250**
isosclerone (**2**)	3.99	10.50	Mean	15.82	29.34	59.17	134.15	266.03
y = 650.00401 x - 4755.59055			Imprecision[a]	7.7	4.1	6.2	7.9	5.6
r = 0.99376			Inaccuracy[b]	5.4	-2.2	-1.4	-10.6	-6.4

a Expressed as coefficient of variation (CV%): (standard deviation/mean) x 100. b Expressed as % difference: [(concentration calculated – concentration expected)/ concentration expected] x 100.

Table S2. Within-day imprecision (CV%) and inaccuracy (%) of results obtained by measuring two QC levels 6 times within one day (n=6).

Peptide	Expected Concentration (µg(mL)	Calculated Cocnentration (µg(mL)	Imprecision (CV%)	inaccuracy (%)
scytalone (**1**)	7	6.6	4.2	-5.7
	20	21.1	2.2	5.5
isosclerone (**2**)	35	34.7	3.1	-0.8
	100	99.8	1.9	-0.2

Table S3. Between-day imprecision (CV%) and inaccuracy (%) of results obtained by measuring two QC levels once a day for five days (n=5).

Peptide	Expected Concentration (µg(mL)	Calculated Concentration (µg(mL)	Imprecision (CV%)	Inaccuracy (%)
scytalone (**1**)	7	7.1	5.6	1.4
	20	20.35	7.6	1.75
isosclerone (**2**)	35	35.8	6.3	2.3
	100	101.8	2.0	1.8

Table S4. Workflow parameters Mzmine 2.

Mass detection	Parameters
mass detector	exact mass
noise level	1.0x10^3
**ADAP chromatogram builder**	**Parameters**
minimum group size	5
group intensity threshold	1.0x10^4
minimum highest intensity	1.0×10^3
m/z tolerance	0.001 m/z - 5 ppm
**Smoothing**	**Parameters**
Filter width	5
**Chromatogram deconvolution**	**Parameters**
algorithm	Wavelets (ADAP)
m/z centre calculation	AUTO
S/N threshold	5
S/N estimator	Intensity window SN
Min feature height	1.0×10^3
Coefficient/area threshold	50
Peak duration range	0.05 - 10
RT wavelet range	0.00 – 0.5
**Deisotoping**	
m/z tolerance	0.001 m/z - 5 ppm
RT tolerance	0.1 absolute (min)
maximum charge	2
representative isotope	most intense
**RANSAC Aligner**	**Parameters**
m/z tolerance	0.001 m/z -5 ppm
RT tolerance	1 min
RT tolerance after correction	0.20
RANSAC iteration	0
Minimum number of points	50%
Threshold value	1
**Gap filling**	**Parameters**
m/z tolerance	0.001 m/z - 5 ppm
intensity tolerance	1%
Ritention time tolerance	0.20 min
